# Pediatric intestinal pseudo‐obstruction found in 3‐year‐old male with Rett‐related mutation of methyl‐CpG binding protein 2

**DOI:** 10.1002/jpr3.70102

**Published:** 2025-10-23

**Authors:** Angela Tran, Namrata Patel‐Sanchez

**Affiliations:** ^1^ Pediatrics, UCSF Benioff Children's Hospital Oakland Oakland California USA; ^2^ Pediatric Gastroenterology UCSF Benioff Children's Hospital Oakland Oakland California USA

**Keywords:** constipation, dysmotility, gastrointestinal disorder, genetic mutation

## Abstract

A 3‐year‐old male with chronic abdominal distention, constipation, and severe malnutrition is diagnosed with pediatric intestinal pseudo‐obstruction (PIPO) after extensive evaluation that excluded mechanical, malabsorptive, metabolic, inflammatory, and infectious causes. Aside from speech delay, he has a normal neurologic exam. Whole exome sequencing reveals a pathogenic methyl‐CpG binding protein 2 (MECP2) variant, suggesting atypical Rett syndrome. Management includes promotility agents and a gastrostomy tube with cyclic feedings of peptide‐based formula, leading to resolution of symptoms. This case highlights the diagnostic complexity of PIPO and the need to consider genetic etiologies, including MECP2‐related disorders, even in patients with mild neurologic findings. Early genetic testing and multidisciplinary care are essential for diagnosis and management in this atypical presentation of Rett syndrome with manifestation of PIPO.

## 1 INTRODUCTION

**Figure 1:**
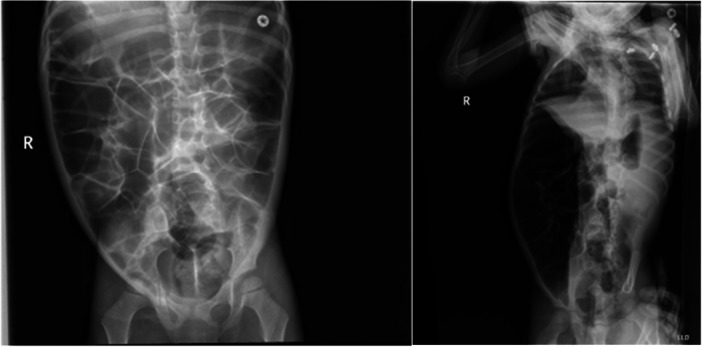
Initial abdominal X‐ray in anteroposterior (left) and lateral decubitus (right) views demonstrating diffuse gaseous distention of the bowel and multiple air‐fluid levels, concerning for gastrointestinal dysmotility.

A 3‐year‐old male with abdominal distention and constipation since birth presents with worsening abdominal distention for 3 weeks. He eats a varied diet though passes hard, infrequent stools without hematochezia, abdominal pain, vomiting or fever. Exam reveals severe malnutrition (body mass index *z*‐score −4.99), short stature (*z*‐score −2.39), speech delay, and a distended abdomen. Labs show normal blood counts, electrolytes, liver enzymes, thyroid function, lipase, and lactate. Abdominal X‐ray shows dilated bowel loops and air‐fluid levels (Figure 1), prompting admission for possible partial bowel obstruction.

## 2 CASE REPORT

A full‐thickness rectal biopsy shows ganglion cells. An esophagram and upper gastrointestinal series with small bowel follow‐through shows prolonged retainment of ingested material, suggestive of delayed gastric emptying, but no obstruction. Further motility testing including gastric emptying scan and manometric studies are not available inpatient at our institution at this time. Renal ultrasound rules out megacystitis. Computed tomography enterography and upper endoscopy are negative for mechanical obstruction though severe small and large bowel dilation is noted. Full‐thickness ileal biopsy is normal. Malnutrition work‐up only shows vitamin D deficiency. Bedside swallow evaluation by occupational therapy reveals functional oropharyngeal skills. Fluoroscopic video swallow study is not recommended at the time. Dietary assessment reveals that while a robust number of calories are offered, oral intake is likely overestimated due to the parent's report of limiting meals when noticing patient's abdominal distention worsening. Stool studies for pathogens, ova and parasites, Helicobacter pylori and human immunodeficiency virus are negative. Malabsorptive studies of stool reducing substances, stool osmolality, stool pH, pancreatic elastase, stool alpha‐1‐antitrypsin, and celiac serologies are normal. Fecal calprotectin is initially elevated, but repeat level is normal. Urine organic and amino acids are abnormal but without a diagnosis. Given inconclusive workup, whole exome is sent. Diagnosis of pediatric intestinal pseudo‐obstruction (PIPO) is made.

Miralax and senna improve stooling, but small intestinal distension remains on X‐ray. A nasogastric tube is placed for nutrition. The tube is also set to low intermittent wall suction after meals, reducing abdominal circumference on one occasion from 52 to 45 cm. Nutritional rehabilitation begins with intact pediatric formula and thiamine. Due to concern for delayed gastric emptying, feeds are given via nasojejunal tube and advanced to full caloric requirement. This is tolerated with less distension and discomfort. Further prokinetics with erythromycin and prucalopride lead to partial improvement. Metoclopramide is trialed but discontinued to minimize medication burden, and due to no significant difference in abdominal distention after the addition. Ultimately, a gastrostomy tube with addition of Farrell bag is placed for venting. Rifaximin is trialed for presumed small intestinal bacterial overgrowth with improvement of abdominal distention. By discharge, the patient has had 13% weight gain receiving peptide‐based formula 1.5 kcal/mL gastric feeds over 20 h with continuous decompression via Farrell bag on senna, erythromycin, and prucalopride.

The patient gains weight rapidly (27% increase) in 1 month with frequent stooling though severely limited oral intake. Whole exome sequencing reveals a novel, pathogenic methyl‐CpG binding protein 2 (MECP2) deletion‐insertion mutation (c.1115_117delinsTG). Due to speech delay and genetic findings, the patient is sent to Rett clinic for support. After calorie reduction, weight gain remains rapid with no increase in oral intake but with worsened stooling after prucalopride is accidentally stopped. Then, he presents with 30% weight loss following cessation of tube feeds due to shipment delay, despite resumption of regular toddler diet and triple medication adherence. Stools are unchanged. After restarting tube feeds, he gains weight steadily but again has limited oral intake. He continues to have speech delay but no regression or seizures.

## 3 DISCUSSION

Pediatric intestinal pseudo‐obstruction is a challenging diagnosis of exclusion that often requires extensive evaluation to rule out structural, endocrine, inflammatory, infectious, malabsorptive, metabolic, and neurogenic causes. His delayed gastric emptying, dilated bowel loops, absence of occluding lesions, and inability to maintain appropriate oral nutrition met criteria for pediatric intestinal pseudo‐obstruction.^1^

In this rare case of PIPO, genetic testing confirmed a MECP2 gene mutation, diagnosing MECP2‐related disorder or atypical Rett syndrome as a possible etiology. MECP2 influences gut motility through the enteric nervous system, in part by regulating neuronal nitric oxide synthase (nNOS).^2^ Peristalsis relies on acetylcholine as an excitatory signal and nitric oxide as the inhibitory signal.^3^ Increased nNOS levels may contribute to dysmotility seen in patients with MECP2 mutations.^2^ Gastrointestinal issues are common in Rett syndrome, MECP2 duplication syndrome, and MECP2‐related disorders, usually alongside neurological symptoms.^4^, ^5^, ^6^ Fluoroscopic video swallow studies can typically diagnose subtle abnormalities in patients with Rett syndrome,^7^ though our dysphagia team did not recommend it at the time of admission. This case is unique in that intestinal pseudo‐obstruction was the first and most prominent feature in a patient with mild neurologic findings. One case series has reported pseudo‐obstruction as the first feature in three patients with MECP2 duplication disorder, but these patients had severe neurodevelopmental disorders.^8^

Another notable aspect is the patient's 30% weight loss after peptide‐based formula cessation, despite ongoing promotility agents and full return of appetite. Although his initial malabsorption workup was negative, we suspect he has decreased ability to absorb intact proteins and fats needing a peptide‐based formula with higher percentage of medium‐chain triglycerides.

## 4 CONCLUSION

This case highlights the importance of considering MECP2‐related disorders in children presenting with symptoms of pediatric intestinal pseudo‐obstruction, even when neurological findings are subtle. Gastrointestinal symptoms may precede neurological ones in atypical Rett syndrome, making early recognition vital and intervention possible with genetic testing, tailored nutritional support, and multidisciplinary care.

## CONFLICT OF INTEREST STATEMENT

The authors declare no conflict of interest.

## ETHICS STATEMENT

Parental informed consent was obtained for the publication of the case details.
